# Lactylation-associated CXCL12-mediated intercellular communication in rheumatic heart disease valves

**DOI:** 10.1016/j.jtauto.2026.100405

**Published:** 2026-10-13

**Authors:** Junyu Zhai, Zheng Zuo, Tao Yan, Fangyu Liu, Jiaxing Liu, Kai Zhu, Hao Lai, Chunsheng Wang, Jun Li

**Affiliations:** Department of Cardiac Surgery, Zhongshan Hospital Fudan University, 180 Fenglin Road, Shanghai, 200032, China

**Keywords:** Rheumatic heart disease, Lactylation, CXCL12, NF-κB, Valve interstitial cells, Single-cell RNA sequencing

## Abstract

**Background:**

Rheumatic heart disease (RHD) is characterized by persistent valvular inflammation and fibrosis. Lactate-driven lysine lactylation may couple metabolic stress to inflammatory transcription, but its relationship with CXCL12-mediated immune recruitment in RHD valves remains unclear.

**Methods:**

Paired aortic and mitral valve single-cell RNA-sequencing data from three patients with RHD were analyzed, yielding 47,237 quality-controlled cells. CXCL12-producing cell populations were identified, and lactylation-related transcriptional states and inflammatory pathways were assessed in CXCL12-high structural cells using complementary scoring and pseudobulk approaches. CellChat was used to infer CXCL12-mediated intercellular communication, with GSE153555 used for external validation. Human valve interstitial cells (VICs) were treated with lactate, the p300 inhibitor A-485, or the NF-κB inhibitor BAY 11-7082. CXCL12/CXCR4-dependent monocyte chemotaxis was evaluated using THP-1 cells, AMD3100, and a CXCL12-neutralizing antibody.

**Results:**

VICs and myofibroblasts were the principal CXCL12-producing cells. Within CXCL12-high structural cells, a higher lactylation-related transcriptional state was associated with enhanced NF-κB and TNF signaling and increased IL6, BIRC3, REL, and TNFAIP3 expression. CellChat supported CXCL12-CXCR4 communication from structural cells to myeloid and lymphoid cells, while external validation confirmed increased CXCL12, IL6, and TNFAIP3 expression in diseased valves. In VICs, lactate increased Pan-Kla, H3K9la, H3K14la, and CXCL12 expression and secretion. A-485 attenuated lactylation-associated CXCL12 induction, whereas BAY 11-7082 suppressed NF-κB activation and CXCL12 expression. Lactate-conditioned medium enhanced THP-1 migration and inflammatory gene expression, which were reduced by pathway inhibition or CXCL12/CXCR4 blockade.

**Conclusion:**

These findings link lactylation-associated metabolic signaling in RHD valve structural cells to NF-κB-dependent CXCL12 production and CXCL12-CXCR4-mediated monocyte recruitment, providing a mechanistic framework for stromal–immune communication in RHD.

## Graphical abstract

**Figure:**
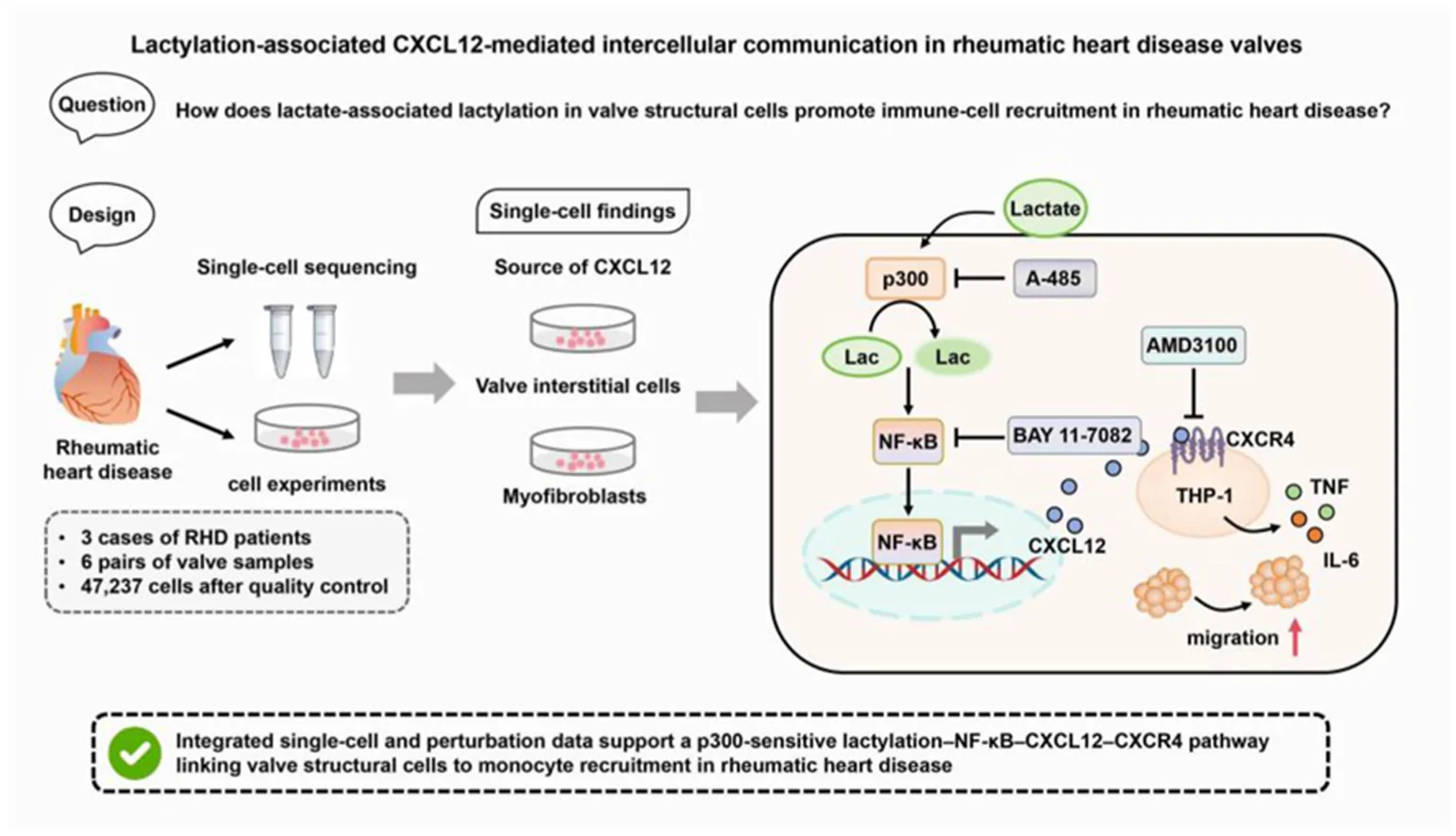

## 1 Introduction

Rheumatic heart disease (RHD) continues to impose a substantial worldwide health concern, predominantly afffecting populations in income-limited nations, where it constitutes a primary cause of cardiovascular morbidity and death among pediatric and young adults [1], [2]. As an autoimmune sequela of Category A streptococcal infection, RHD manifests as progressive valvular damage and cardiac dysfunction, driven by chronic inflammation and fibrotic remodeling [3]. Despite the availability of surgical interventions such as valve replacement, the need for long-term anticoagulation and the compromised quality of life after surgery underscore the urgent need for deeper mechanistic insights and novel therapeutic strategies [4].

Lactate is both a metabolic product and a signaling metabolite. Lysine lactylation, including histone lactylation, can couple glycolytic stress to transcriptional regulation [5], [6]. Inflamed and fibrotic tissues are therefore plausible sites in which lactate accumulation alters stromal-cell behavior. RHD valves contain activated immune and mesenchymal populations, but whether lactylation participates in their communication has not been defined.

Given the close interplay between metabolic reprogramming and inflammatory cell trafficking in chronic valvular injury, we next focused on CXCL12 as a potential mediator linking stromal responses to immune-cell recruitment. CXCL12 is a homeostatic chemokine with potent chemotactic and cell-retention functions, acting predominantly through CXCR4, while ACKR3 provides context-dependent modulation of CXCL12 signaling [7]. The CXCL12–CXCR4/ACKR3 axis regulates immune-cell trafficking and tissue repair and can also influence glycolytic metabolism [8], [9], [10]. CXCL12–CXCR4 signaling has also been implicated in atrial inflammation, fibrosis, and atrial fibrillation, further supporting its relevance to cardiovascular inflammatory remodeling [11]. Notably, preliminary interrogation of our RHD single-cell dataset localized CXCL12 expression predominantly to valve structural cells, particularly VICs and MyoFBs. This cell-specific pattern provided a rationale for investigating whether lactylation-associated signaling converges on the CXCL12 axis to regulate stromal–immune communication in RHD.

In this study, we integrated paired-valve single-cell transcriptomic analysis with targeted validation experiments to characterize CXCL12-producing cell populations and lactylation-associated inflammatory states in RHD valves. The primary novelty lies in linking valve structural-cell metabolic alterations with CXCL12-mediated stromal–immune communication at single-cell resolution and extending these observations through functional validation. This integrated approach provides new insight into the cellular basis of persistent valvular inflammation in RHD and highlights a potential metabolic–immune regulatory pathway for future therapeutic investigation.

## 2 Materials and methods

### 2.1 Human valve samples

Paired mitral valve (MV) and aortic valve (AV) specimens were obtained from three patients with clinically and histologically confirmed RHD who underwent valve-replacement surgery, yielding six samples (AV1/MV1, AV2/MV2, and AV4/MV4). The study was approved by the Institutional Review Board of Zhongshan Hospital Fudan University (B2021-793R). All participants provided written informed consent, and the study complied with the Declaration of Helsinki.

### 2.2 Single-cell RNA-sequencing data processing

The six 10x Genomics expression matrices comprised 49,971 cell barcodes. Analyses were performed in R 4.4.2 (https://www.r-project.org/) with Seurat 5.5.0 (https://satijalab.org/seurat/). Cells were retained when 200≤nFeature_RNA≤4,000, 200≤nCount_RNA≤10,000, and mitochondrial transcripts were≤20%. After quality control, 47,237 cells remained. Counts were log-normalized with a scale factor of 10,000, 2000 highly variable genes were selected, samples were integrated by reciprocal principal component analysis, and principal components 1-30 were used for UMAP and graph-based clustering at resolution 0.4. The random seed was 20260730.

Sixteen clusters were consolidated into six major cell types after review of canonical markers and cluster-level expression patterns: VICs, MyoFBs, vascular endothelial cells, myeloid cells, lymphocytes, and mast/B cells. Annotation was based on marker evidence rather than a single automated label. Sample-specific cell counts, quality metrics, UMAPs, marker expression, and cell-type proportions were inspected to assess integration and possible batch effects.

### 2.3 Definition of CXCL12-high structural cells

CXCL12 expression was evaluated across all annotated cell types. Because VICs and MyoFBs were the main sources, subsequent state analyses were restricted to their combined 36,781 cells. CXCL12-high cells were defined by log-normalized CXCL12 expression above the pooled 75th percentile (1.778) within VICs and MyoFBs. Robustness was assessed at the 70th (1.618) and 80th (1.950) percentiles. Differential-expression concordance across thresholds was quantified by Pearson correlation.

### 2.4 Lactylation-related gene set and scoring

The LRG list was derived from the published 332-gene compilation provided in Table S1. After trimming whitespace and collapsing duplicate entries for CDV3 and SFPQ, 330 unique genes remained; 303 were detected in the single-cell matrix. We evaluated the resulting lactylation-associated transcriptional state with Seurat AddModuleScore, UCell, and sample-by-cell-type pseudobulk ssGSEA using GSVA 2.0.7 (Bioconductor). These expression scores were interpreted as transcriptional correlates and not as direct measurements of lysine lactylation.

CXCL12-high and CXCL12-low cells were compared within each sample and cell type. For analysis within CXCL12-high VICs and MyoFBs, LRG-low and LRG-high states were defined as the lower and upper quartiles, respectively, within each sample-by-cell-type block; the middle 50% was retained for audit but excluded from the extreme-state contrast.

### 2.5 Differential expression, pathway analysis, and core genes

Pseudobulk counts were generated for each sample, cell type, and LRG state and analyzed with DESeq2 1.46.0 (https://bioconductor.org/packages/DESeq2/), blocking by biological sample and cell type. Benjamini-Hochberg false-discovery rates (FDRs) below 0.05 were considered significant. NF-κB, TNF, and IL-17 pathway scores were calculated by ssGSEA. An exploratory WGCNA analysis was also performed on pseudobulk profiles; because no module remained associated with LRG-high status after multiple-testing correction, WGCNA was treated as a robustness analysis rather than the basis for the main claim.

IL6, BIRC3, REL, TNFAIP3, and ZC3H12A were prespecified as core inflammatory candidates based on their prior network position, direction in the cell-level LRG-high comparison, and membership in NF-κB/TNF-related programs. The earlier protein-protein interaction analysis used STRING (https://string-db.org/; *Homo sapiens*; minimum interaction score 0.4) and Cytoscape 3.10.2 with MCODE. The revised analysis reports donor-blocked effect sizes and FDRs for each candidate and does not label a gene significant when its FDR exceeds 0.05.

### 2.6 Cell-cell communication

Cell-cell communication was inferred with CellChat 2.2.0.9001 (https://github.com/jinworks/CellChat) and CellChatDB.human. A merged analysis used the complete Secreted Signaling database, and sample-level CXCL-subset models were fitted separately for all six samples. CXCL12-high VICs and MyoFBs were treated as candidate senders and myeloid and lymphoid cells as receivers. CXCL12-CXCR4 and CXCL12-ACKR3 probabilities, bootstrap P values, receptor expression, and paired LRG-high versus LRG-low probabilities were evaluated.

### 2.7 External transcriptomic validation

GSE153555 was used as an independent valve-disease cohort. The dataset contains 15 biological donors (five normal, five aortic stenosis, and five aortic insufficiency), each represented by two sequencing libraries. Technical libraries were merged within donor before analysis; the 30 libraries were not treated as independent biological replicates. Disease groups were compared with controls for CXCL12, the five core genes, and NF-κB, TNF, and IL-17 ssGSEA scores, with FDR correction. Because GSE153555 is not an RHD cohort and does not measure lactylation, it was used only to test directional reproducibility in diseased valves.

### 2.8 Cell culture and perturbation experiments

Human valvular interstitial cells (VICs; SNP-H359; Sunncell) and THP-1 human monocytes (TIB-202; ATCC) were maintained at 37°C in a humidified atmosphere containing 5% CO_2_. VICs were cultured in high-glucose DMEM (11965092; Gibco) supplemented with 10% fetal bovine serum (FBS; 10099141; Gibco) and 1% penicillin–streptomycin (15140122; Gibco), whereas THP-1 cells were maintained in RPMI-1640 medium (11875093; Gibco) supplemented with 10% FBS.

VICs were treated with sodium L-lactate (20 mM; L7022; Sigma-Aldrich) for 24 h. For inhibition of p300/CBP activity, cells were pretreated with A-485 (HY-107455; MedChemExpress) for 1 h before lactate exposure. NF-κB signaling was inhibited using BAY 11-7082 (1 μM; HY-13453; MedChemExpress), either alone or as a 30-min pretreatment before lactate stimulation.

Total RNA was extracted from cells using the FastPure Cell/Tissue Total RNA Isolation Kit (Vazyme, Nanjing, China) according to the manufacturer's instructions. cDNA was synthesized using HisyGo All-in-One RT Red SuperMix for qPCR (+gDNA Wiper; RT333-01; Vazyme). Quantitative real-time PCR was performed using SupRealQ Ultra Hunter SYBR qPCR Master Mix (U+; Q713-02; Vazyme) with gene-specific primers. Relative gene expression was calculated using the 2^^−ΔΔCt^ method, with GAPDH as the internal reference. Primer sequences are listed in Table 1.

**Table 1:** Primer sequences used for qRT-PCR.

Gene	Direction	Primer sequence (5′–3′)
CXCL12	Forward	TAATGAACGCCAAGGTCGTG
CXCL12	Reverse	TTCTCCAGGTACTCCTGAATC
IL-6	Forward	TAATGAACTCCTTCTCCACAAGC
IL-6	Reverse	GTTACATGTCTCCTTTCTCAGG
TNF-α	Forward	TAATGAGCACTGAAAGCATGATCC
TNF-α	Reverse	TGCTACAACATGGGCTACAG

Western blotting was performed using primary antibodies against Pan-Kla (HUABIO, Cat. No. HA722037; 1:2000), CXCL12 (Cell Signaling Technology, Cat. No. 3740; 1:1000), H3K9la (PTM BIO, Cat. No. PTM-1419RM; 1:2000), H3K14la (PTM BIO, Cat. No. PTM-1414RM; 1:2000), phospho-NF-κB p65 (Cell Signaling Technology, Cat. No. 3033; 1:1000), NF-κB p65 (Cell Signaling Technology, Cat. No. 8242; 1:1000), phospho-IκBα (Abcam, Cat. No. ab133462; 1:1000), and IκBα (Affinity Biosciences, Cat. No. AF5002; 1:2000). GAPDH (Proteintech, Cat. No. 81640-5-RR; 1:10000) and histone H3 (Abcam, Cat. No. ab1791; 1:5000) were used as loading controls, as appropriate. Membranes were subsequently incubated with HRP-conjugated goat anti-rabbit IgG secondary antibody (Bioss, Cat. No. bs-0295G-HRP; 1:3000). Protein bands were visualized by chemiluminescence and quantified using ImageJ.

### 2.9 THP-1 chemotaxis

Conditioned media were collected from treated VICs in drug-free low-serum medium. THP-1 migration was assessed using 24-well Transwell inserts with 8.0-μm polycarbonate membranes (Corning, Cat. No. 3422). Experimental conditions included control-conditioned medium, lactate-conditioned medium, lactate + A-485-conditioned medium, lactate + BAY 11-7082-conditioned medium, and lactate-conditioned medium combined with THP-1 pretreatment with the CXCR4 antagonist AMD3100 (10 μM, 30 min; MedChemExpress, Cat. No. HY-10046). Additional groups included lactate-conditioned medium containing a CXCL12-neutralizing antibody (R&D Systems, Cat. No. MAB310) and recombinant human CXCL12/SDF-1α (R&D Systems, Cat. No. 350-NS) as a positive chemotactic control. Migrated cells were counted in three randomly selected microscopic fields. IL6 and TNF mRNA levels in THP-1 cells were subsequently quantified by RT-qPCR.

### 2.10 Statistical analysis and replication

The single-cell discovery cohort comprised three biological donors with paired AV and MV samples. Sample- or donor-level units were used for inferential analyses whenever possible. Paired comparisons used two-sided paired tests; multi-group bioinformatics analyses used the model-specific tests described above, with Benjamini-Hochberg FDR correction. CellChat edge significance was based on its bootstrap procedure.

Cell experiments were repeated independently three times and are shown as mean ± SD. Multi-group comparisons used one-way analysis of variance followed by Tukey-adjusted comparisons. A two-sided P value below 0.05 was considered significant.

## 3 Results

### 3.1 A paired-valve single-cell atlas identifies six major cell types

Quality control retained 47,237 of 49,971 cell barcodes. RPCA integration and clustering identified 16 clusters that were consolidated into VICs (32,206 cells), MyoFBs (4,575), myeloid cells (6,806), lymphocytes (2,101), mast/B cells (905), and vascular endothelial cells (644). Canonical markers supported each annotation, and VICs were the most abundant population in every sample (Fig. 1 and Fig. S1).

**Fig. 1:**
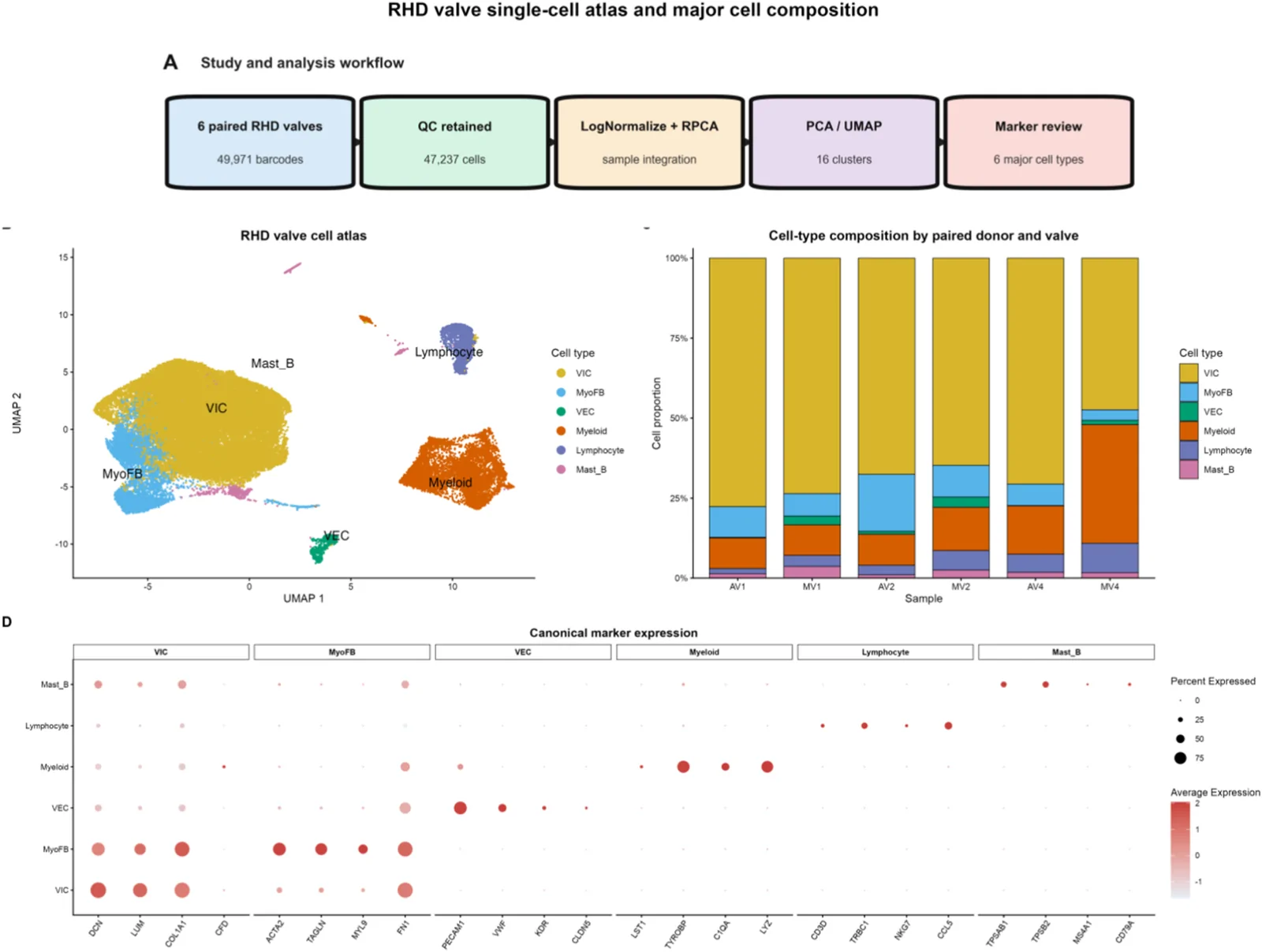
RHD valve single-cell atlas and major cell composition. A, Study workflow and retained cell numbers. B, UMAP of six annotated cell types. C, Cell-type composition for each paired valve sample. D, Canonical marker expression supporting the annotations.

### 3.2 CXCL12 is produced predominantly by VICs and MyoFBs

CXCL12 positivity and mean normalized expression were highest in MyoFBs (55.34%; 1.089) and VICs (46.72%; 0.873), whereas expression was substantially lower in immune populations. The q75 threshold identified sample-specific CXCL12-high fractions in both structural populations (Fig. 2). MV samples showed numerically higher CXCL12-high fractions than paired AV samples, but the donor-paired differences were not significant for VICs (MV-AV = 0.0509, FDR = 0.423) or MyoFBs (MV-AV = 0.0638, FDR = 0.363).

**Fig. 2:**
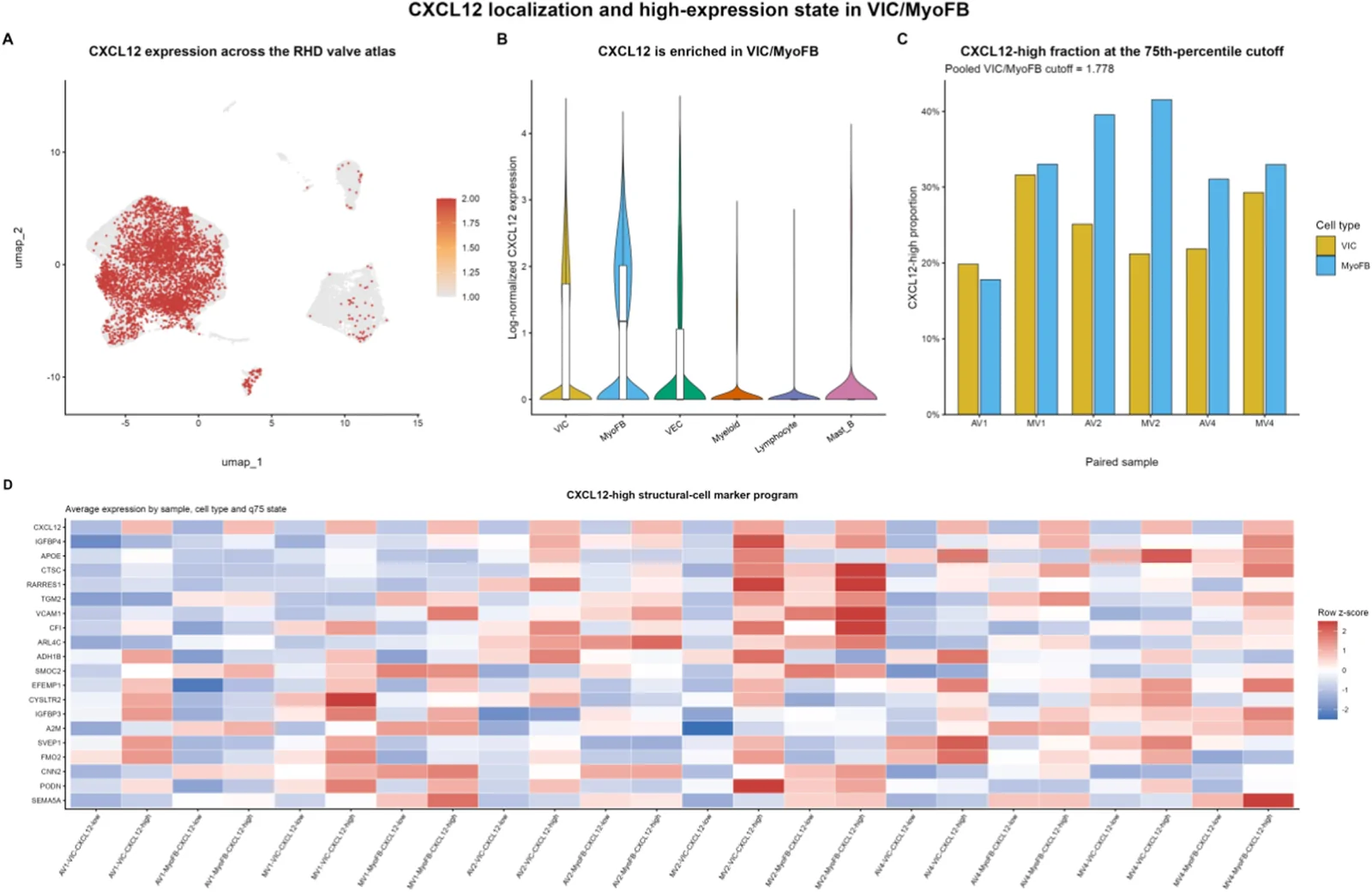
CXCL12 localization and high-expression state in VICs and MyoFBs A, CXCL12 expression across the atlas. B, Expression by cell type. C, CXCL12-high fractions at the pooled 75th-percentile cutoff. D, Structural-cell marker program across sample, cell type, and CXCL12 state.

Threshold sensitivity analysis supported the definition. Differential-expression log-fold changes at q70 and q80 were highly correlated with q75 results (r = 0.997 and r = 0.996, respectively; Fig. S2). These findings establish VICs and MyoFBs as the main CXCL12 sources.

### 3.3 Relationship between CXCL12-high status and global LRG scores

The three LRG scoring methods showed good concordance at the sample-by-cell-type level (Pearson r = 0.791–0.904). However, CXCL12-high status was not consistently associated with higher global LRG scores. Paired comparisons showed that the differences between groups did not reach statistical significance for AddModuleScore, UCell, or pseudobulk ssGSEA (all FDR > 0.05). At the cellular level, the correlations between CXCL12 expression and LRG scores were also weak (VICs: ρ = 0.071; MyoFBs: ρ = −0.089; Fig. 3).

**Fig. 3:**
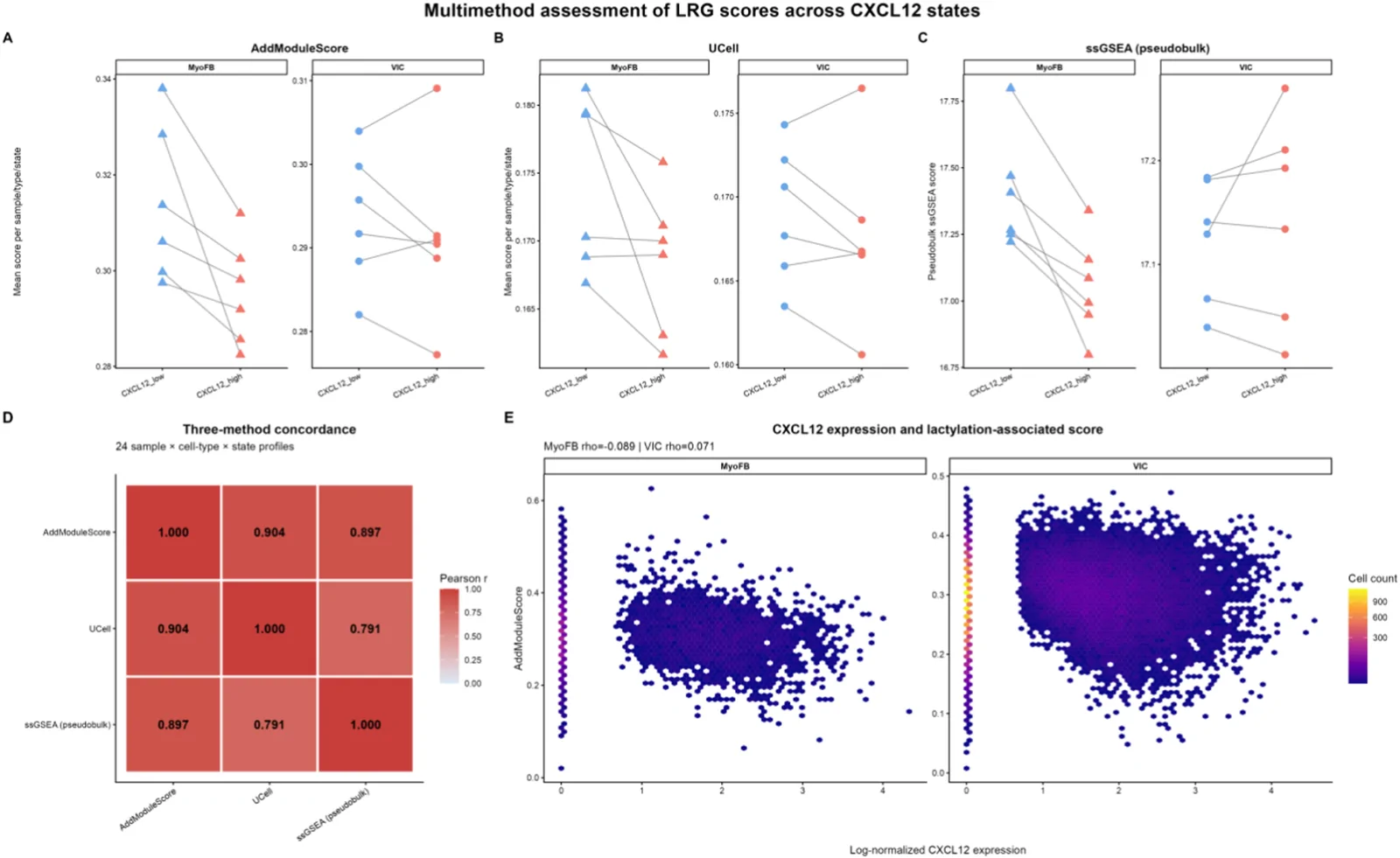
Multimethod assessment of lactylation-related gene scores across CXCL12 states A-C, Paired CXCL12-low and CXCL12-high comparisons using AddModuleScore, UCell, and pseudobulk ssGSEA. D, Concordance among scoring methods. E, Weak cell-level correlations between CXCL12 expression and AddModuleScore.

These findings suggest that CXCL12 expression status and the global LRG transcriptional signature are not fully concordant and may reflect related but distinct aspects of the underlying biology. Removal of CXCL12, IL6, and core NF-κB-related genes had minimal impact on the LRG scores (Fig. S3).

### 3.4 An LRG-high state within CXCL12-high structural cells is associated with NF-κB and TNF programs

Within CXCL12-high VICs and MyoFBs, the upper LRG-score quartile showed higher NF-κB (high-low = 0.229, FDR = 0.00629) and TNF (high-low = 0.0801, FDR = 0.00629) ssGSEA scores. The IL-17 difference was not significant (high-low = 0.0432, FDR = 0.410; Fig. 4). Donor- and cell-type-blocked pseudobulk analysis showed higher expression of IL6 (logCPM difference 1.811, FDR = 0.00632), BIRC3 (1.681, FDR = 0.00632), REL (1.411, FDR = 0.00643), and TNFAIP3 (0.927, FDR = 0.0135). ZC3H12A changed in the same direction but did not meet the FDR threshold (0.592, FDR = 0.0653).

**Fig. 4:**
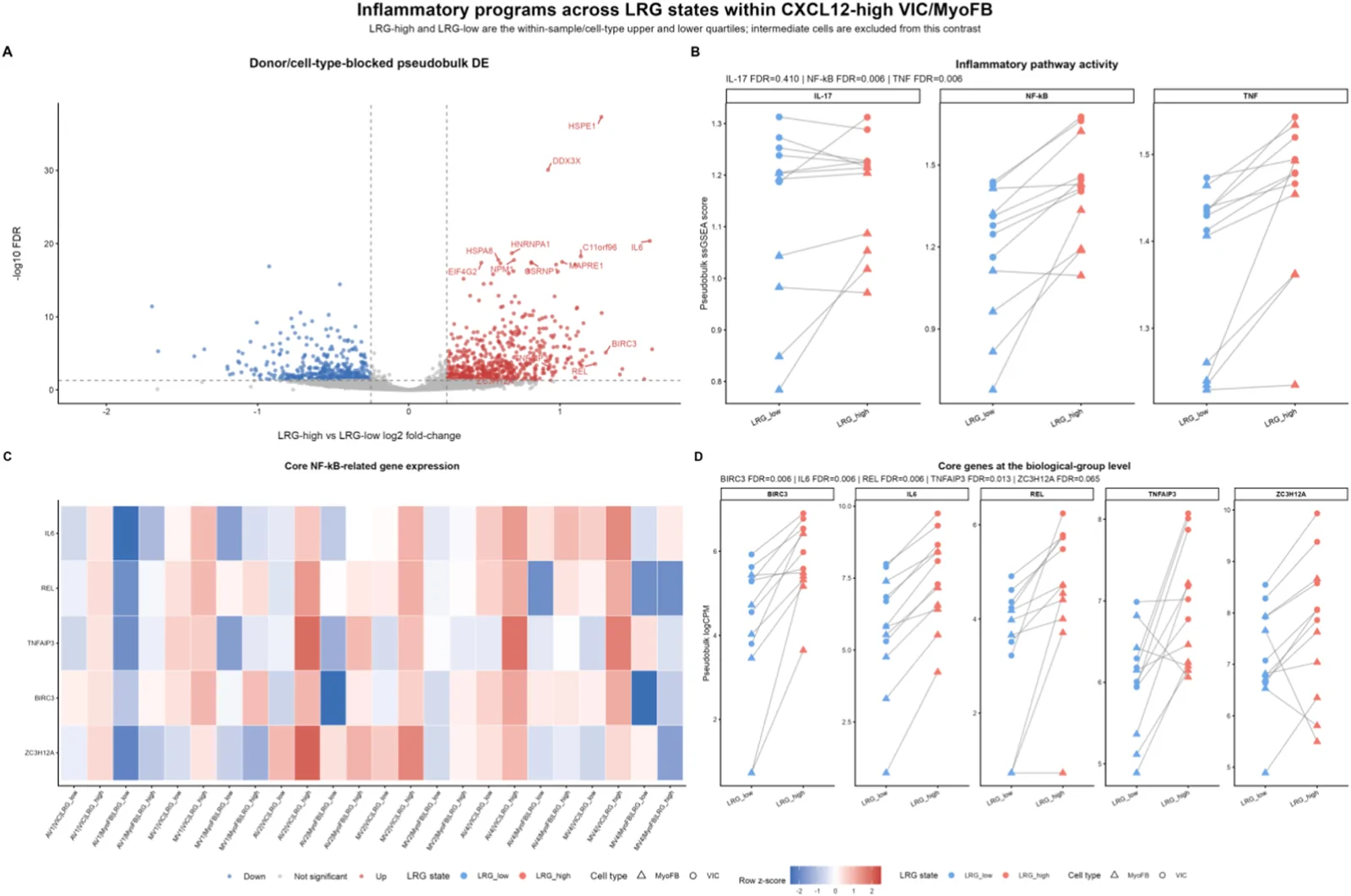
Inflammatory programs across LRG states within CXCL12-high VICs and MyoFBs A, Donor- and cell-type-blocked pseudobulk differential expression. B, Paired inflammatory pathway scores. C, Core NF-kappaB-related gene expression across blocks. D, Pseudobulk expression of five candidate genes. LRG-high and LRG-low denote upper and lower within-block quartiles.

Pseudobulk WGCNA primarily captured cell-type structure, and no module remained significantly associated with LRG-high status after correction (Fig. S4).

### 3.5 CXCL12-CXCR4 links structural cells to immune cells

The merged CellChat model included 838 overexpressed ligand-receptor pairs and 349 significant communication edges. CXCL12-CXCR4 communication was consistently inferred from VICs and MyoFBs to myeloid and lymphoid cells, with bootstrap P < 0.01 for all displayed edges. CXCR4 was detected in 54.72% of myeloid cells and 88.20% of lymphocytes, whereas ACKR3 was uncommon (7.79% and 0.81%, respectively; Fig. 5 and Fig. S5).

**Fig. 5:**
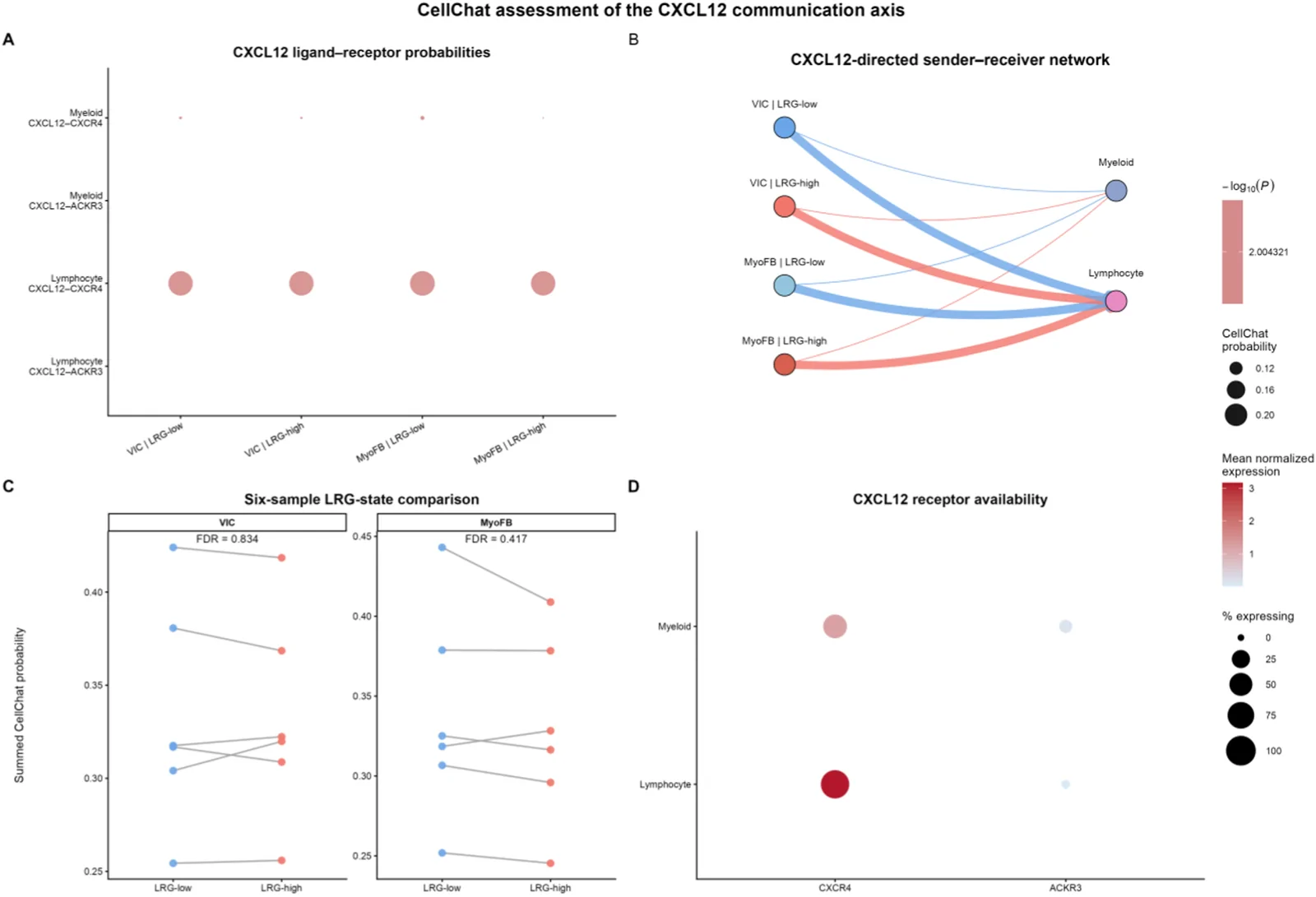
CellChat assessment of the CXCL12 communication axis A, CXCL12 ligand-receptor probabilities. B, Structural-cell-to-immune-cell network. C, Six-sample paired comparison of LRG states. D, CXCR4 and ACKR3 availability in myeloid cells and lymphocytes.

Sample-level comparisons did not show stronger CXCL12 communication in the LRG-high state for VICs (FDR = 0.834) or MyoFBs (FDR = 0.417). The data therefore support the presence of a structural-cell-to-immune-cell CXCL12-CXCR4 axis but not amplification of this axis by the LRG-high transcriptional state.

### 3.6 External valve data validate expression changes in key genes

In the external valve disease cohort GSE153555, donor-level aggregation analysis showed that the expression levels of CXCL12, IL6, and TNFAIP3 were significantly higher in diseased valves, with log2 fold changes of 1.700 (FDR = 1.35 × 10^-10), 3.164 (FDR = 2.88 × 10^-4), and 1.025 (FDR = 1.27 × 10^-5), respectively (Fig. 6 and Fig. S6). These findings provide further support from an independent valve disease cohort for the CXCL12 and related inflammatory gene expression signatures identified in this study.

**Fig. 6:**
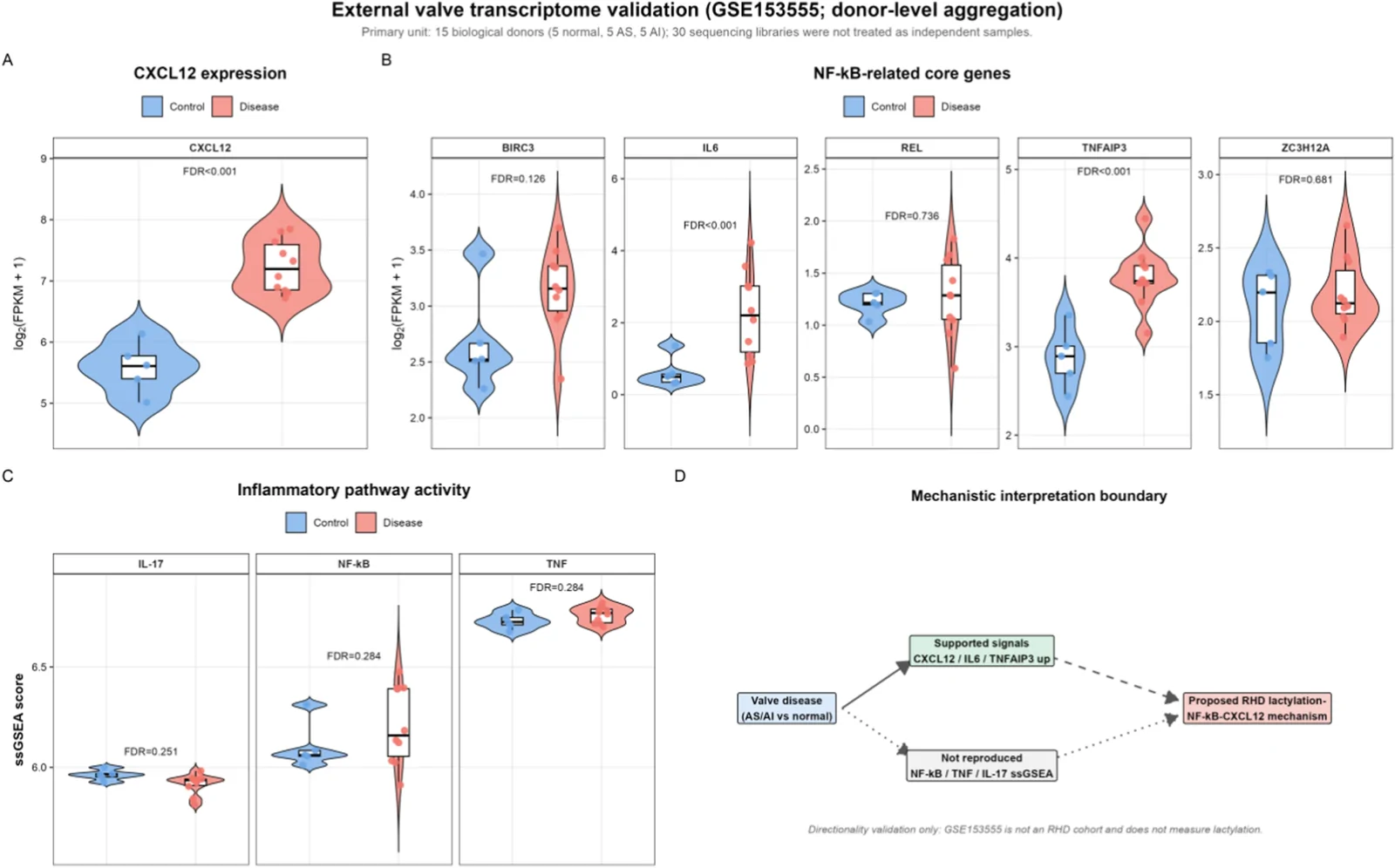
External valve transcriptome validation in GSE153555 A, Donor-level CXCL12 expression. B, Five candidate NF-kappaB-related genes. C, Inflammatory pathway scores. D, Interpretation boundary. Fifteen biological donors were analyzed after within-donor aggregation of technical libraries.

### 3.7 Lactate increases lactylation and CXCL12 expression in VICs

Lactate treatment significantly increased CXCL12 mRNA expression and CXCL12 secretion in VICs. Consistently, Western blotting showed increased Pan-Kla, H3K9la, H3K14la, and CXCL12 protein levels after lactate exposure. Pretreatment with A-485 markedly attenuated these lactate-induced changes, although histone lactylation remained partially elevated relative to control levels (Fig. 7).

**Fig. 7:**
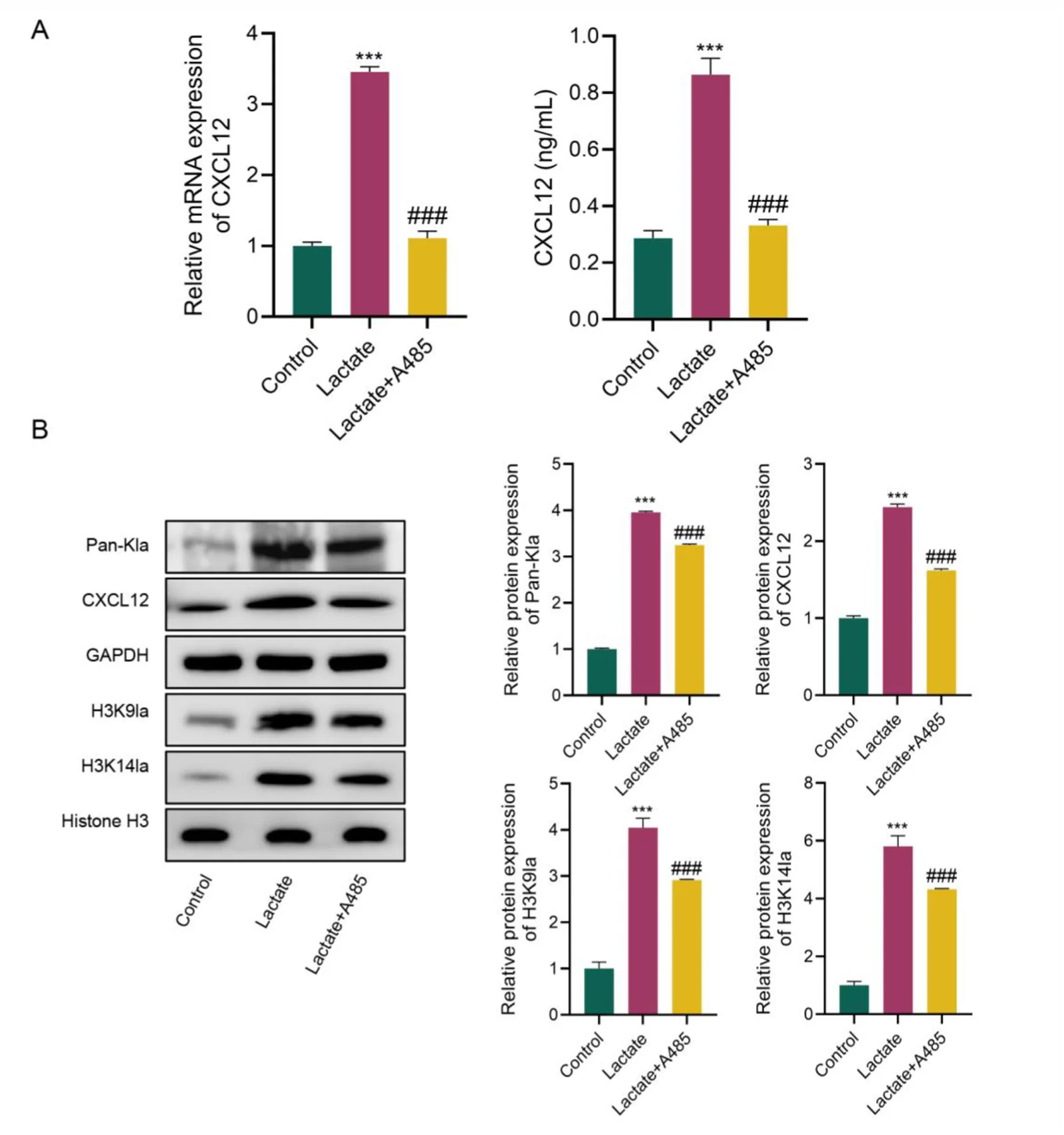
Lactate-induced lactylation and CXCL12 expression in human VICs A, CXCL12 mRNA expression. B, CXCL12 secretion measured by ELISA. C, Representative Western blots and densitometric analyses of Pan-Kla, CXCL12, H3K9la, and H3K14la. A-485 attenuated lactate-induced changes. Data are mean ± SD from three independent experiments. ***P < 0.001 vs. control; ###P < 0.001 vs. lactate.

### 3.8 NF-κB inhibition attenuates lactate-induced CXCL12 expression

Lactate treatment increased p-p65 and p-IκBα levels while reducing IκBα, consistent with activation of the NF-κB pathway. Pharmacological inhibition with BAY 11-7082 markedly attenuated these signaling changes and reduced lactate-induced CXCL12 protein expression. Notably, H3K9la remained elevated relative to control despite NF-κB inhibition (Fig. 8A).

**Fig. 8:**
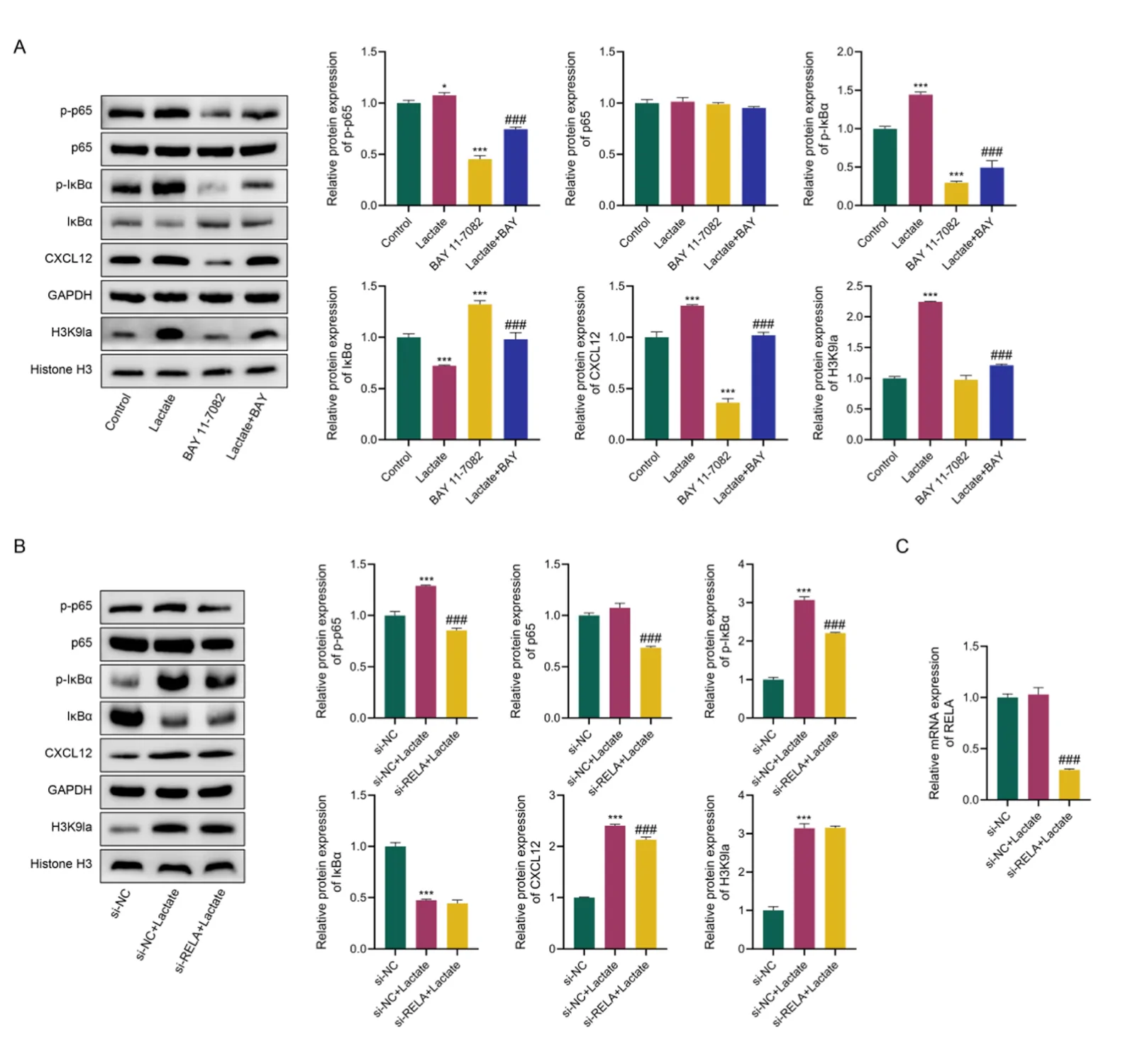
NF-κB inhibition attenuates lactate-induced CXCL12 expression in VICs. A, VICs were treated with lactate with or without BAY 11-7082. Representative Western blots and quantification show p-p65, p65, p-IκBα, IκBα, CXCL12, and H3K9la levels. BAY 11-7082 attenuated lactate-induced NF-κB activation and CXCL12 expression. B, VICs were transfected with si-NC or si-RELA and exposed to lactate. RELA knockdown reduced p65, p-p65, p-IκBα, and CXCL12 levels, with minimal effect on H3K9la. C, RT-qPCR confirmed effective RELA knockdown in VICs. Data are presented as mean ± SD. *P < 0.05, **P < 0.01, ***P < 0.001 vs. control or si-NC group; #P < 0.05, ##P < 0.01, ###P < 0.001 vs. lactate-treated or si-NC + lactate group.

To further verify the involvement of NF-κB, RELA was silenced in VICs. RELA knockdown significantly reduced RELA mRNA expression and decreased p65, p-p65, p-IκBα, and CXCL12 levels in lactate-treated cells, while H3K9la remained largely unchanged (Fig. 8B and C). Together, these pharmacological and genetic interventions support NF-κB as an important downstream mediator linking lactate-associated histone lactylation to CXCL12 induction.

### 3.9 CXCL12–CXCR4 signaling contributes to monocyte chemotaxis and inflammatory activation

Conditioned medium from lactate-treated VICs significantly increased THP-1 migration compared with control-conditioned medium. This promigratory effect was markedly attenuated when VICs were treated with A-485 or BAY 11-7082, and was also reduced by CXCR4 blockade with AMD3100 or neutralization of CXCL12. Conversely, recombinant human CXCL12 elicited a comparable increase in THP-1 migration (Fig. 9A).

**Fig. 9:**
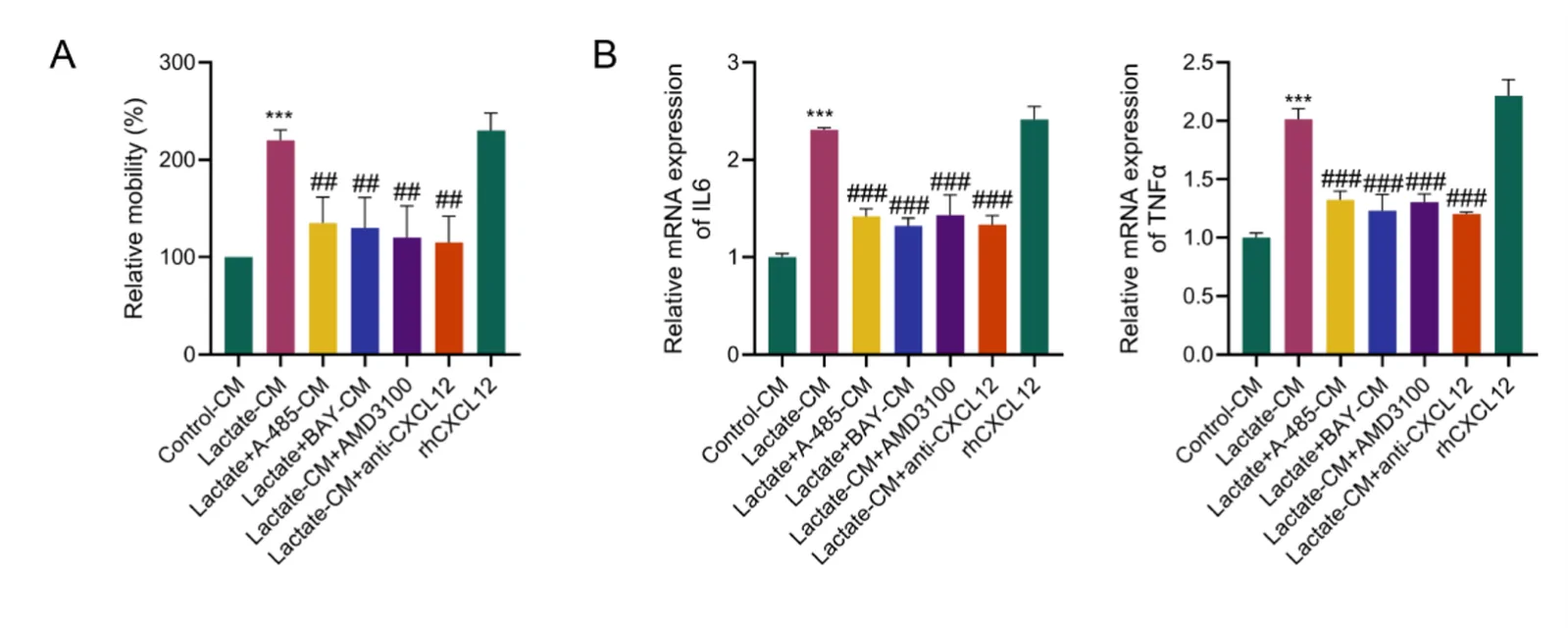
CXCL12-CXCR4 signaling contributes to THP-1 chemotaxis and inflammatory activation A, Relative THP-1 migration in response to VIC-conditioned media and pathway blockade. B, THP-1 IL6 and TNF mRNA. AMD3100 and a CXCL12-neutralizing antibody attenuated responses to lactate-conditioned medium, while recombinant CXCL12 served as a positive control. Data are presented as mean ± SD. ***P < 0.001 vs. control; ##P < 0.01 and ###P < 0.001 vs. lactate.

Consistent with the migration response, lactate-conditioned medium significantly increased IL6 and TNF mRNA expression in THP-1 cells. These inflammatory responses were attenuated by A-485, BAY 11-7082, AMD3100, and CXCL12-neutralizing antibody, whereas recombinant CXCL12 reproduced the induction of IL6 and TNF (Fig. 9B).

## 4 Discussion

This study delineates a cellular and functional framework linking metabolic regulation in valve structural cells to inflammatory cell recruitment in RHD. Single-cell analysis localized CXCL12 predominantly to VICs and MyoFBs, while pathway analysis within CXCL12-high structural cells connected lactylation-related transcriptional states with NF-κB/TNF activity. These observations are consistent with the emerging view that RHD is sustained not only by immune-cell infiltration but also by active interactions among stromal, endothelial, and immune compartments within chronically inflamed valves. Recent reviews of RHD pathobiology emphasize persistent valvular inflammation, VIC activation, myofibroblast expansion, and extracellular-matrix remodeling as central features of progressive structural damage.

Recent reviews of RHD pathobiology emphasize persistent valvular inflammation, VIC activation, myofibroblast expansion, and extracellular-matrix remodeling as central features of progressive structural damage [12], [13]. In this study, a major finding of the single-cell analysis is that CXCL12 expression and the LRG score represent related but distinct biological features. CXCL12-high VICs and MyoFBs did not simply correspond to cells with uniformly higher global LRG scores. Instead, within CXCL12-high structural cells, the LRG-high state was associated with stronger NF-κB and TNF pathway activity and higher expression of IL6, BIRC3, REL, and TNFAIP3. This pattern suggests that lactylation-related transcription may be most informative when interpreted in a defined cellular and inflammatory context rather than as a direct surrogate for biochemical protein lactylation. Histone lactylation was originally identified as a metabolite-responsive chromatin modification capable of coupling cellular lactate availability to transcriptional regulation. More recently, lactate-driven histone lactylation has also been shown to alter fibroblast phenotypes and extracellular-matrix programs in fibrotic tissue, supporting a broader role for metabolic-epigenetic signaling in stromal remodeling.

The human VIC experiments provide functional support for this metabolic-epigenetic link. Lactate increased Pan-Kla, H3K9la, H3K14la, CXCL12 mRNA, CXCL12 protein, and secreted CXCL12, whereas A-485 attenuated these responses. p300/CBP has been implicated in lactylation-associated chromatin regulation, and A-485 is a selective catalytic inhibitor of p300/CBP [14]. Accordingly, the present findings are appropriately interpreted as evidence for p300-sensitive lactylation. The downstream signaling experiments further strengthen the sequence of events: lactate increased p-p65 and p-IκBα and reduced IκBα, whereas BAY 11-7082 attenuated NF-κB activation and CXCL12 induction. In parallel, RELA knockdown reduced p65-dependent signaling and CXCL12 expression while leaving H3K9la comparatively preserved. Together, the pharmacological and genetic interventions are consistent with NF-κB acting downstream of lactate-associated histone lactylation and contributing to CXCL12 induction in VICs.

CXCL12-CXCR4 signaling provides a biologically plausible route by which these structural-cell changes could influence the immune compartment. CXCL12 is a well-established chemotactic and retention signal, and CXCR4 has important roles in leukocyte trafficking, progenitor-cell homing, tissue repair, and cardiovascular inflammation [15]. The CXCL12-CXCR4 axis has also been implicated in fibrotic remodeling across multiple organs, where it can influence inflammatory-cell recruitment and fibroblast behavior [16]. In the present study, CellChat predicted CXCL12-CXCR4 communication from VICs and MyoFBs toward myeloid and lymphoid cells, consistent with the high prevalence of CXCR4 in these recipient populations. Functional experiments supported this prediction: conditioned medium from lactate-treated VICs increased THP-1 migration and induced IL6 and TNF expression, whereas CXCR4 blockade with AMD3100 or neutralization of CXCL12 substantially attenuated these responses. Recombinant CXCL12 reproduced the promigratory and inflammatory effects.

The candidate-gene and external-validation results provide additional support for an inflammatory context surrounding this pathway. IL6, BIRC3, REL, and TNFAIP3 were increased in the LRG-high state within CXCL12-high structural cells, linking the identified transcriptional state to regulators and effectors of NF-κB/TNF signaling. In the independent GSE153555 valve-disease cohort, CXCL12, IL6, and TNFAIP3 were also increased in diseased valves. These observations are consistent with current models of rheumatic valvular fibrosis, in which persistent inflammatory signaling is associated with VIC activation, myofibroblast expansion, and extracellular-matrix remodeling. In this context, the enrichment of NF-κB/TNF-related programs in LRG-high CXCL12-high structural cells may reflect an inflammatory state that links metabolic-epigenetic alterations in VICs/MyoFBs to the remodeling of the valvular microenvironment [17], [18]. Because GSE153555 is not an RHD-specific cohort, its value lies in demonstrating reproducibility of selected valve-disease-associated signals rather than establishing disease specificity.

From a translational perspective, the results nominate a metabolically responsive stromal-immune circuit that may be relevant to persistent valvular inflammation. Established RHD still lacks a disease-modifying therapy capable of reversing advanced valve remodeling, and surgery remains the definitive intervention for severe structural disease [17], [19]. The lactylation-NF-κB-CXCL12-CXCR4 pathway therefore identifies several experimentally testable intervention points, including modulation of pathological lactate/lactylation, NF-κB signaling, and CXCL12-CXCR4-mediated immune recruitment. However, CXCL12-CXCR4 and NF-κB also have essential physiological roles in host defense, hematopoietic trafficking, tissue repair, and cell survival. Any therapeutic application would therefore require careful assessment of tissue specificity, dosing, delivery strategy, and long-term safety rather than systemic pathway suppression.

The study has important limitations. The discovery cohort contained only three patients and paired end-stage surgical valves, with no healthy valve comparator. Single-cell expression scores cannot measure protein lactylation, and pseudobulk aggregation only partly addresses donor-level uncertainty. GSE153555 represents non-RHD valve disease and provides directional rather than disease-specific validation. The in vitro system lacks the full valve microenvironment, and no animal or prospective clinical validation was performed. Raw and processed source files, code, uncropped blots, and numerical figure data should accompany the resubmission to facilitate independent evaluation.

## 5 Conclusion

This study identifies VICs and MyoFBs as major CXCL12-producing cells in RHD valves and supports a lactylation–NF-κB–CXCL12–CXCR4 signaling framework linking metabolic alterations in valve structural cells to immune-cell recruitment. By integrating single-cell transcriptomics with targeted functional experiments, our findings provide a cellular and mechanistic basis for stromal–immune communication in RHD and highlight metabolic–inflammatory crosstalk as a potentially important contributor to persistent valvular inflammation. Further validation in larger RHD cohorts and valve-relevant in vivo models is warranted.

## CRediT authorship contribution statement

**Junyu Zhai:** Writing – review & editing, Writing – original draft, Visualization, Validation, Supervision, Software, Resources, Project administration, Methodology, Investigation, Funding acquisition, Formal analysis, Data curation, Conceptualization. **Zheng Zuo:** Writing – review & editing, Visualization, Validation, Software, Project administration, Investigation, Formal analysis, Data curation, Conceptualization. **Tao Yan:** Writing – review & editing, Visualization, Validation, Software, Project administration, Investigation, Formal analysis, Data curation. **Fangyu Liu:** Writing – review & editing, Visualization, Supervision, Resources, Methodology, Formal analysis, Data curation. **Jiaxing Liu:** Writing – review & editing, Visualization, Supervision, Resources, Methodology, Formal analysis, Data curation. **Kai Zhu:** Writing – review & editing, Visualization, Supervision, Resources, Methodology, Formal analysis, Data curation. **Hao Lai:** Writing – review & editing, Visualization, Supervision, Resources, Methodology, Formal analysis, Data curation. **Chunsheng Wang:** Writing – review & editing, Writing – original draft, Visualization, Validation, Supervision, Software, Resources, Project administration, Methodology, Investigation, Funding acquisition, Formal analysis, Data curation, Conceptualization. **Jun Li:** Writing – review & editing, Writing – original draft, Visualization, Validation, Supervision, Software, Resources, Project administration, Methodology, Investigation, Funding acquisition, Formal analysis, Data curation, Conceptualization.

## Ethics declaration

Written informed consent to take part in the study and to publish the article has been obtained from all participants or their legal representatives. The privacy rights of participants have been observed

This study was performed in compliance with relevant laws, regulatory frameworks and guidelines where the research took place. This study was approved by the Zhongshan Hospital Fudan University. (Approval No. B2021-793R).

## Ethics approval and consent to participate

The study was approved by the Institutional Review Board of Zhongshan Hospital Fudan University (B2021-793R). All participants provided written informed consent before surgery, and the study was conducted in accordance with the Declaration of Helsinki.

## Consent for publication

Not applicable.

## Availability of data and material

The single-cell RNA-sequencing and external transcriptomic datasets analyzed in this study are publicly available from the corresponding repositories, with accession information provided in the Methods. The analytical procedures are described in detail to facilitate reproducibility. Raw experimental data are available from the corresponding author upon reasonable request.

## Funding statement

Not applicable.

## Declaration of competing interests

The authors declare that they have no known competing financial interests or personal relationships that could have appeared to influence the work reported in this paper.

## Data Availability

Data will be made available on request.

## References

[1] Ou Z., Yu D., Liang Y., Wu J., He H., Li Y., He W., Gao Y., Wu F., Chen Q. (2022). Global burden of rheumatic heart disease: trends from 1990 to 2019. Arthritis Res. Ther..

[2] Ralph A.P., Noonan S., Wade V., Currie B.J. (2021). The 2020 Australian guideline for prevention, diagnosis and management of acute rheumatic fever and rheumatic heart disease. Med. J. Aust..

[3] Lv M., Jiang S., Liao D., Lin Z., Chen H., Zhang J. (2022). Global burden of rheumatic heart disease and its association with socioeconomic development status, 1990-2019. Eur. J. Prev. Cardiol..

[4] Li N., Zhu L., Zhou H., Zheng D., Xu G., Sun L., Gao J., Shao G. (2020). miRNA-1183-targeted regulation of Bcl-2 contributes to the pathogenesis of rheumatic heart disease. Biosci. Rep..

[5] Fan H., Yang F., Xiao Z., Luo H., Chen H., Chen Z., Liu Q., Xiao Y. (2023). Lactylation: novel epigenetic regulatory and therapeutic opportunities. Am. J. Physiol. Endocrinol. Metab..

[6] Sun Z., Song Y., Li J., Li Y., Yu Y., Wang X. (2023). Potential biomarker for diagnosis and therapy of sepsis: lactylation. Immun. Inflamm. Dis..

[7] Mehrpouri M. (2022). The contributory roles of the CXCL12/CXCR4/CXCR7 axis in normal and malignant hematopoiesis: a possible therapeutic target in hematologic malignancies. Eur. J. Pharmacol..

[8] Liu X., Wang H., Ni W., Dong X., Zheng M., Chang W. (2020). Global lactylome reveals lactylation-dependent mechanisms underlying CXC motif chemokine ligand 12 expression in pulmonary endothelium during acute respiratory distress syndrome. MedComm.

[9] Luker K.E., Luker G.D. (2022). The CXCL12/CXCR4/ACKR3 signaling axis regulates PKM2 and glycolysis. Cells.

[10] Dichtl S., Lindenthal L., Zeitler L., Behnke K., Schlosser D., Strobl B., Scheller J., El Kasmi K.C., Murray P.J. (2021). Lactate and IL6 define separable paths of inflammatory metabolic adaptation. Sci. Adv..

[11] Liu P., Sun H., Zhou X., Wang Q., Zheng Q. (2021). CXCL12/CXCR4 axis as a key mediator in atrial fibrillation via bioinformatics analysis and functional identification. Cell Death Dis..

[12] Yeow S., Frost H., Porrello E.R., Steer A., Voges H.K. (2026). Valve biology and rheumatic heart disease pathogenesis. Nat. Cardiovasc. Res..

[13] Liu Z., Liu R., Liang J., Wang M., Wang M., Zhang H., Jiang W. (2025). Chronic mitral valve fibrosis in rheumatic heart disease: from immune trigger to inflammatory and mechanical progression. J. Am. Heart Assoc..

[14] Lasko L.M., Jakob C.G., Edalji R.P., Qiu W., Montgomery D., Digiammarino E.L., Hansen T.M., Risi R.M., Frey R., Manaves V., Shaw B., Algire M., Hessler P., Lam L.T., Uziel T., Faivre E., Ferguson D., Buchanan F.G., Martin R.L., Torrent M., Chiang G.G., Karukurichi K., Langston J.W., Weinert B.T., Choudhary C., de Vries P., Van Drie J.H., McElligott D., Kesicki E., Marmorstein R., Sun C., Cole P.A., Rosenberg S.H., Michaelides M.R., Lai A., Bromberg K.D. (2017). Discovery of a selective catalytic p300/CBP inhibitor that targets lineage-specific tumours. Nature.

[15] Doring Y., Pawig L., Weber C., Noels H. (2014). The CXCL12/CXCR4 chemokine ligand/receptor axis in cardiovascular disease. Front. Physiol..

[16] Wu X., Qian L., Zhao H., Lei W., Liu Y., Xu X., Li J., Yang Z., Wang D., Zhang Y., Zhang Y., Tang R., Yang Y., Tian Y. (2023). CXCL12/CXCR4: an amazing challenge and opportunity in the fight against fibrosis. Ageing Res. Rev..

[17] Mai T.A., Marchandot B., Hmadeh S., Nguyen H.T.T., Pham H.M., Nguyen Q.N., Schini-Kerth V., Pibarot P., Morel O. (2026). Rheumatic heart disease: inflammation, immune pathways, valve remodeling, and the promise of SGLT2 inhibitors. JACC, Basic Transl. Sci..

[18] Kim L., Kim D.K., Yang W.I., Shin D.H., Jung I.M., Park H.K., Chang B.C. (2008). Overexpression of transforming growth factor-beta 1 in the valvular fibrosis of chronic rheumatic heart disease. J Korean Med Sci.

[19] Vervoort D., Yilgwan C.S., Ansong A., Baumgartner J.N., Bansal G., Bukhman G., Cannon J.W., Cardarelli M., Cunningham M.W., Fenton K., Green-Parker M., Karthikeyan G., Masterson M., Maswime S., Mensah G.A., Mocumbi A., Kpodonu J., Okello E., Remenyi B., Williams M., Zuhlke L.J., Sable C. (2023). Tertiary prevention and treatment of rheumatic heart disease: a national heart, lung, and blood institute working group summary. BMJ Glob. Health.

