# Supplementary material for: Lactylation-associated CXCL12-mediated intercellular communication in rheumatic heart disease valves
Source: J Transl Autoimmun. 2026 Oct 13;13:100405. doi: 10.1016/j.jtauto.2026.100405 (PMC13734818; doi:10.1016/j.jtauto.2026.100405)
Supplement: Supplementary file 1 [file mmc2.docx]

**Supplementary Figure Legends**

Figure S1. Quality control and sample integration for the six RHD valve single-cell datasets.

Figure S2. Sensitivity of CXCL12-high classification to the 70th-, 75th-, and 80th-percentile thresholds.

Figure S3. Sensitivity analysis after auditing overlap between the LRG set and CXCL12, IL6, and core NF-kappaB genes.

Figure S4. GSEA and WGCNA robustness analyses. No WGCNA module remained associated with LRG-high status after multiple-testing correction.

Figure S5. Overview of CellChat secreted-signaling results across the six samples.

Figure S6. External-validation quality control and sensitivity analyses for GSE153555.
